# Novel Surfactant-Induced MWCNTs/PDMS-Based Nanocomposites for Tactile Sensing Applications

**DOI:** 10.3390/ma15134504

**Published:** 2022-06-27

**Authors:** Anindya Nag, Nasrin Afsarimanesh, Suresh Nuthalapati, Mehmet Ercan Altinsoy

**Affiliations:** 1 Faculty of Electrical and Computer Engineering, Technische Universität Dresden, 01062 Dresden, Germany; suresh.nuthalapati@tu-dresden.de (S.N.); ercan.altinsoy@tu-dresden.de (M.E.A.); 2 Centre for Tactile Internet with Human-in-the-Loop (CeTI), Technische Universität Dresden, 01069 Dresden, Germany; 3 School of Civil and Mechanical Engineering, Curtin University, Perth, WA 6102, Australia; nasrin.afsarimanesh@curtin.edu.au

**Keywords:** MWCNTs, PDMS, nanocomposites, doping, SDS

## Abstract

The paper presents the use of surfactant-induced MWCNTs/PDMS-based nanocomposites for tactile sensing applications. The significance of nanocomposites-based sensors has constantly been growing due to their enhanced electromechanical characteristics. As a result of the simplified customization for their target applications, research is ongoing to determine the quality and quantity of the precursor materials that are involved in the fabrication of nanocomposites. Although a significant amount of work has been done to develop a wide range of nanocomposite-based prototypes, they still require optimization when mixed with polydimethylsiloxane (PDMS) matrices. Multi-Walled Carbon Nanotubes (MWCNTs) are one of the pioneering materials used in multifunctional sensing applications due to their high yield, excellent electrical conductivity and mechanical properties, and high structural integrity. Among the other carbon allotropes used to form nanocomposites, MWCNTs have been widely studied due to their enhanced bonding with the polymer matrix, highly densified sampling, and even surfacing throughout the composites. This paper highlights the development, characterization and implementation of surfactant-added MWCNTs/PDMS-based nanocomposites. The prototypes consisted of an optimized amount of sodium dodecyl sulfonate (SDS) and MWCNTs mixed as nanofillers in the PDMS matrix. The results have been promising in terms of their mechanical behaviour as they responded well to a maximum strain of 40%. Stable and repeatable output was obtained with a response time of 1 millisecond. The Young’s Modulus of the sensors was 2.06 MPa. The utilization of the prototypes for low-pressure tactile sensing applications is also shown here.

## 1. Introduction

With exponential growth in the use of nanotechnology in the field of material science, there is a constant requirement to optimize the performances of micro- and nanosized prototypes. While the process of synthesis of the sensors is pretty much standardized, the same for characterization and application varies greatly with the variation in materials. The sequential simulation and prototyping of the sensors allow for the combination of materials. With a wide spectrum of available nanomaterials [1], there is simultaneous growth in the efficiency of the sensors. From the fabrication point of view, it is pivotal to consider the safety of the researchers as the process of conjugation of some of the nanomaterials with other processed materials leads to the formation of toxic by-products [2]. Regarding the application of the sensors, the demand for reusing each prototype with an assurance of consistently high robustness, longevity and sensitivity is an issue that needs to be resolved in academic as well as industrial scenarios. Even though the sensors developed and utilized in the controlled laboratory environment perform with high sensitivity for target applications, prototyping those sensors for commercial purposes is still not done for a wide range of prototypes.

After the popularization of semiconducting sensors for commercial purposes at the beginning of the 1990s [3,4], single-crystal silicon-based prototypes were employed for various applications. Certain attributes like small size, excellent signal-to-noise ratio, and an ability to operate under different environmental conditions [5,6] have led the researchers to employ them in different kinds of industrial [7,8,9] and environmental [10,11,12] applications. These prototypes have been formed using the conventional technique of microelectromechanical systems (MEMS) involving the process of lithography [13,14]. Although these sensors have been found to serve their purposes, their rigidity and high cost of fabrication and degradation of their responses over time led researchers to opt for alternative forms of the sensor. Researchers have since moved on to develop flexible sensors with materials that have high tensile strength and mechanical integrity. 

The fabrication processes that are used to form the flexible sensors are called printing techniques [15]. As a replacement, these sensors used organic, inorganic and synthetic polymers to form the substrates. Some of the common polymers used to form the flexible sensors are polydimethylsiloxane (PDMS) [16,17], polyethylene terephthalate (PET) [18,19], polyimide (PI) [20,21] and polyaniline (PI) [22,23]. Nowadays, conductive polymers like poly (3,4-ethylenedioxythiophene): poly(styrene sulfonate) (PEDOT: PSS) [24,25] are also being used to maintain the electrical conductivity and mechanical flexibility of the resultant prototypes. Among them, the performance of PDMS has been extraordinary to form wearable sensors due to its biocompatibility, hydrophobicity, ability to form homogenous interfacial bonds with nanofillers, and resistance in its response to a change in ambient conditions. Similar to the polymers, the electrodes of the flexible sensors have also been formed using a wide range of nanomaterials. This variation is inclusive of both the structure and the type of the nanomaterial. While some of the common physiochemical structures include nanotubes [26], nanobeads [27], nanoparticles [28], nanoribbons [29] and quantum dots [30], the materials can be broadly classified into carbon allotropes [31,32] and metallic nanomaterials [33,34]. The former includes Carbon Nanotubes (CNTs) [35,36], graphene [37,38] and graphite [39,40], and the latter involves some common metallic nanoparticles and nanowires. Out of these mentioned carbon-based nanomaterials, CNTs have been widely used to form flexible sensors due to their outstanding electrical conductivity, high mechanical flexibility and integrity, high strength, and excellent durability. Among CNTs, both single-walled carbon nanotubes (SWCNTs) and multi-walled carbon nanotubes (MWCNTs) have been used equally for sensing applications. This paper showcases the use of MWCNTs/PDMS-based composites for tactile sensing applications.

The use of CNTs-based nanocomposites has been very effective for tactile sensing applications [41,42] due to their high sensitivity and constant and fast response. They offer highly flexible sensors with high electrical and mechanical properties imparted from the CNTs. PDMS has been used for many biomedical applications due to its low fabrication cost, flexibility and impermeability toward water [43]. The nanocomposites have been widely considered for sensing applications due to their outstanding physical properties. These nanocomposites have found a wide range of potential applications in different fields [44,45,46,47]. These sensors have been predominantly used for wearable sensing applications [48,49]. Although quite a significant amount of work has been done in this sector, one of the primary concerns that remains involve the homogeneity of the CNTs when mixed as nanofillers. Normally, CNTs are mixed commonly with organic polymers by mechanical force or ultrasonication process. The disadvantages associated with the mechanical mixing of CNTs is their improper dispersion in the polymer matrix. In addition, the ultrasonication process of the CNTs destroys the mechanical structure of the nanotubes, causing a change in their properties. It is thus necessary to develop techniques that assist in the proper dispersion of nanotubes in order to maintain the structural integrity of the nanotubes as well as achieve high conductivity of the resultant nanocomposite. 

This paper presents dopant-functionalized MWCNTs/PDMS nanocomposites to increase the sensitivity for long-term purposes. The doping of nanomaterials has always imparted additional advantages over the pre-doped devices [50]. The concentration of doping places a crucial role in the functional capabilities of the device. Sodium dodecyl sulfonate (SDS) is one of the common surfactants that has been used with CNTs for better dispersion in common solvents [51,52]. It increases the hydrophilicity of CNTs by attaching themselves to the hydrophobic part of the nanotube and others with any solvent for dissolution [53]. SDS was added to the nanocomposites of CNT/PDMS at different proportions to study the electromechanical characteristics of the resultant nanocomposites. These nanocomposites have also been used for tactile sensing applications.

The novelty of this work can be presented in two parts. Even though MWCNTs-based prototypes have been formed using SDS as the surfactant, this paper shows an enhancement in the mechanical characteristics of the sensors as compared to the ones previously researched in this area [54,55]. Secondly, none of the presented work has thus utilized the surfactant-assisted prototypes for tactile sensing. Some of the advantages of these surfactant-assisted tactile sensors are the low cost of fabrication, easy fabrication process, high resolution, low power consumption, high stability, and repeatability of the results. The prototypes were developed using PDMS as the polymer matrix and carboxylic acid-functionalized MWCNTs as the nanofillers. SDS was used as surfactants to increase the overall electromechanical properties of these sensors. A simple fabrication process was carried out via manual mixing of the processed materials to form flexible sensors. With the optimized values of MWCNTs and SDS in the polymer matrix, the Young’s Modulus of the sensors was 2.06 MPa. When the prototypes were employed for tactile sensing applications, they showed high stability and repeatability in the responses. The sensors displayed oscillatory changes in the resistance values when a subsequent pressure was applied to the sensory area.

The paper has been structured as follows. Following the introduction described in Section 1, the details of the fabrication of the doped nanocomposite structure are presented in Section 2. Section 3 provides the experimental setup and highlights the instruments used for characterization and experimental purposes. Section 4 shows the output of the sensors; the conclusion is drawn in the paper’s Section 5.

## 2. Fabrication of the Nanocomposites

**Figure 1:**
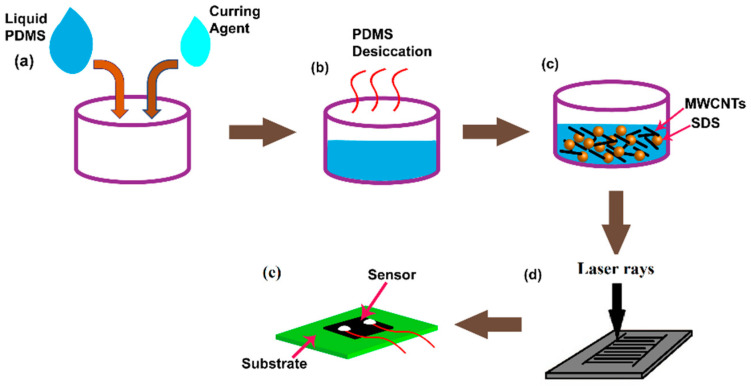
Schematic diagram of steps involved in the process of fabrication of MWCNTs/SDS/PDMS-based sensors.

In these experiments, MWCNTs (COOH-MWCNTs) from Sigma-Aldrich (773840-100G) with a purity of >99.9% were employed as the nanofillers in the PDMS matrix (SYLGARD^®^ 184, Silicon Elastomer Base). Carboxylic acid (-COOH)-functionalized MWCNTs (Aldrich, 773840-100G) were chosen for these experiments. The inner and outer diameters of these MWCNTs were 4.5 nm ± 0.5 nm and 10 nm ± 1 nm, respectively. The length of the nanotubes was between 3–6 µm. The functionalized MWCNTs were considered over SWCNTs due to their better interfacial bonding and dispersion capability in the polymer matrix [56,57,58]. Sodium dodecyl sulfonate (3599286-100G) was used as the surfactant to increase the dispersing capability of CNTs, which eventually affected the electrical and mechanical properties of the nanocomposite. Figure 1 shows a schematic diagram of the steps followed in this process. Poly (methyl methacrylate) (PMMA) substrate was taken as the template to form the samples. The substrate was formed using PDMS, where the pre-polymer and the cross-linking agent of the PDMS were mixed in a ratio of 10:1. The mixture was then desiccated for an hour to remove any air bubbles present inside the polymer. The PDMS layer was then cured inside the oven at 80 °C for 8 h. Another layer of PDMS was formed on top of it with the same ratio (10:1) between the pre-polymer and the curing agent. CNTs were then mixed with the polymer at different weight percentages (wt. %), following which the surfactant SDS was added. Each time, the samples were desiccated in a vacuum and cured inside the oven at 80 °C for 8 h. Different values of wt. % of SDS were considered for a single value of wt. % of CNT in order to determine the precise effect of the surfactants.

**Figure 2:**
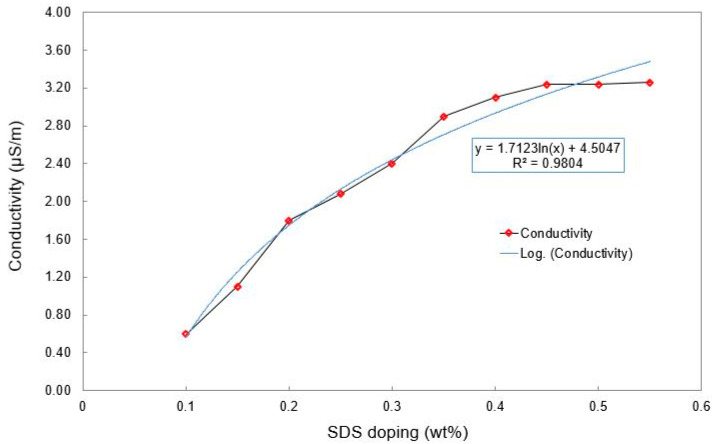
Change in the electrical conductivity with SDS doping at a fixed CNT wt. %.

Table 1 shows the individual values of wt. % of CNTs and the corresponding SDS mixed with them to form the electrodes. The mixing of SDS with the MWCNTs/PDMS nanocomposites was done manually for a short time. Ultra-sonication of the composing structure was avoided to maintain the structural integrity of the MWCNTs. The differences in the electrical conductivities before and after the addition of the MWCNTs were measured. Based on the differences, the value of CNTs was fixed at 0.6 wt. % and the wt. % of SDS were varied to detect the electrical conductivity. It is evident from Figure 2 that the conductive nature of the electrodes increases almost linearly with the increase in the wt. % of SDS. However, the doping with the surfactant does not affect the conductivity after a value of 0.5 wt. % of SDS has been added to the MWCNTs.

**Figure 3:**
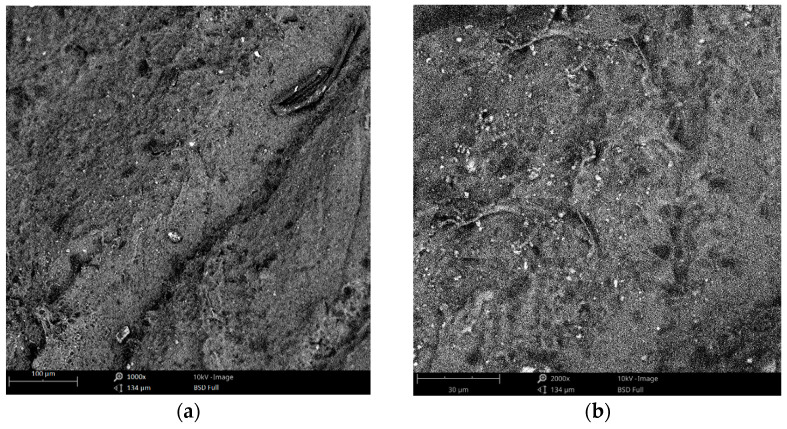
SEM images of the MWCNTs/SDS/PDMS composites at magnifications of (**a**) 1000× and (**b**) 2000×. The white and black colours represent the presence of surfactants and MWCNTs, respectively.

The conductivity is not affected because once the adsorption of the CNTs on the surfactant becomes saturated, any further addition of surfactant cannot increase the hydrophilicity. At the same time, the hydrophobic nature of the MWCNTs would still persist in a self-polymerization process, forming a micelle [59]. As such, a value of 0.6 weight percentage of MWCNTs and 0.5 weight percentage of SDS was arranged to be mixed with PDMS to form the final nanocomposites. The samples were then again desiccated and cured to solidify the nanocomposites. Interdigitated electrodes were formed on the samples using a laser cutting system (Model: OLS 6.75 CO_2_ laser system, laser spot diameter: 150 microns). Four pairs of electrode fingers were formed, where the distance between the two consecutive fingers was 120 microns. The surface area of the final prototypes was 16 mm^2^. Figure 3a,b show the SEM image of the CNT/PDMS nanocomposites doped with SDS. The image presents the top view of the prototypes. Uniform mixing was obtained for these nanocomposites, as is noticeable in the image.

## 3. Experimental Setup

Due to the flexibility of the prototypes, the electromechanical characteristics of the fabricated devices were carried out with respect to the applied strain. The Scanning Electron Microscopic (SEM) images were carried out by using a Zeiss Supra 55VP instrument. The operating voltage and mode to obtain the SEM images were 5 keV and high-vacuum mode, respectively. The samples were mounted on a stub using conductive tapes. The working distance was 6.7 mm. The characterization and experiments were conducted in the laboratory at a fixed temperature (25 °C) and humidity (45%) conditions. The tensile-strength measurement was carried out using an EXCEED E42 Universal Test System from MTS. The crosshead velocity to apply the stress on the prototypes was chosen to be 0.28 mm/s. The response of these sensors was found to be more or less linear, with a fracture point at a strain value of 68%. 

The reduction in the fracture point of the nanocomposites is likely due to two factors. Firstly, even though the tensile strength of CNTs is very high, the presence of other processed materials like PDMS and SDS reduced the overall strain fracture point. Secondly, as the strain was applied perpendicular to the interdigitated electrode fingers, the anisotropic geometry of the electrodes led to the tearing of the sample when the strain value reached the fracture point. The output of the sensors was detected using HIOKI IM 3536 LCR High Precision Tester. While one end of the impedance analyzer was connected to the sensors using Kelvin probes, the other end was connected to a computer via USB-USB cable to collect the data. The data was collected in Microsoft Office Suite using an automatic data acquisition algorithm. The response time of the sensors was 1 millisecond. An average of three readings was considered in order to obtain the final output. This experiment used an alternating signal of 1 V RMS and a frequency of 1 kHz as input values.

## 4. Performance of the Nanocomposite-Based Sensors

**Figure 4:**
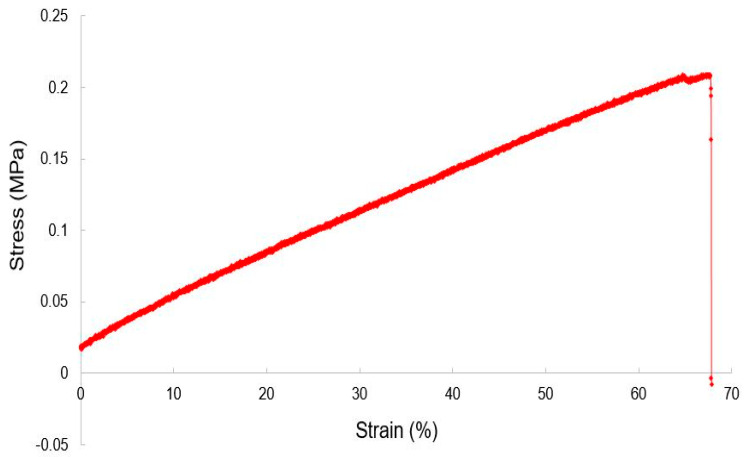
Load-extension relation for the nanocomposite-based sensors.

**Figure 5:**
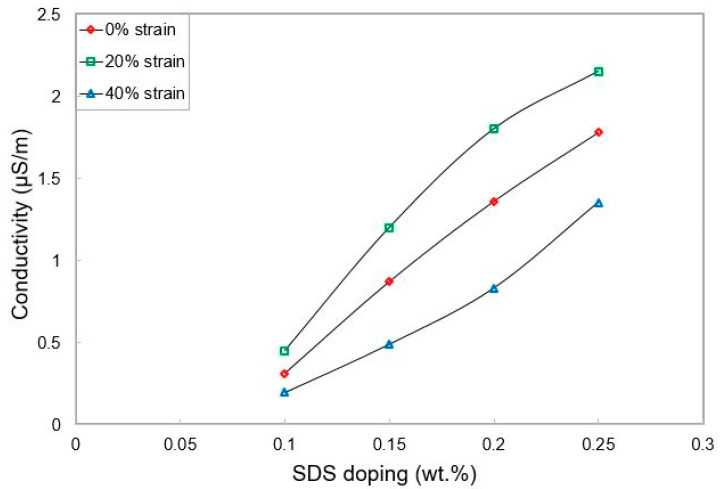
Response of the sensors to the change in conductivity with respect to strain.

Figure 4 shows the response of these sensors for load-extension cycles. After the prototypes were clamped to avoid their sidewise movement, the stress applied to the sensors was perpendicular to the electrodes. On the basis of Hooke’s law, the Young’s Modulus of these prototypes was calculated to be 2.06 MPa. This value is slightly more than pure PDMS [60], i.e., less than 2 MPa. This change can be attributed to the presence of nanofillers in the polymer matrix. The change in the orientation of the MWCNTs and SDS in the polymer matrix led to changes in the corresponding responses under the applied strain. The samples were flexible enough to be used for tactile sensing applications. Figure 5 shows the response of the sensors in terms of conductivity for different strains. It is seen that the conductivity decreases with the increase in strain for a particular doped value. This is because as the exerted strain is increased on the sensors, the connectivity between the nanofillers decreases. This subsequently reduces the amount of current tunnelled between the MWCNTs and SDS, thus reducing the overall resistance value. The change in the electrical conductivity with respect to strain can be attributed to a few factors, including the matrix composition, the electrical conductivity of the nanophase, and the grain size of the matrix [61]. The presence of MWCNTs, in this case, assists in obtaining superior electrical conductivity due to their ultra-fast transportation of electrons. The composition of matrices is significant as the differences in compositions will subsequently change the overall conductivity values.

The segregation of the elements occurring in the matrix-second phase interfaces, which in this case is the non-covalent bonding between the MWCNTs and SDS surfactants, will subsequently tune the effective electrical properties. The dynamic nature of local compositions in the matrix decides the overall interfacial charge density in terms of free-electron transport. The matrix grain, referring to the specific type of nanofillers, decides the overall conductivity based on the Hall–Petch strengthening effect. The smaller value size of the nanofillers increases the density at the boundaries. This decreases the electron mean free path, thus increasing the scattering of electrons. Other factors affecting the change in conductivity with respect to doping and strain are the concentration and geometry of the nanofillers and the geometry of the composites.

**Figure 6:**
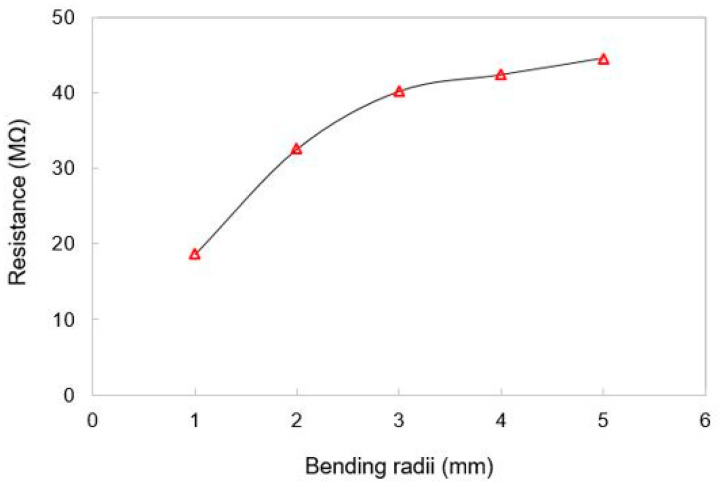
Response of the sensors for different bending radii.

Figure 6 shows the response of sensors with respect to the bending radii. It is observed that the samples respond well to each of the bending radii ranging from 1 mm to 5 mm. This experiment was conducted with an outward bending of the sensors. The resistance values of the prototypes increased with an increase in the bending radii due to a subsequent increase in the connectivity between the MWCNTs and the SDS. When the radius of the curvature increases, the connectivity between individual MWCNTs and SDS particles increases, thus decreasing the effective transport path for the current flow. The prototypes were then tested for continuous bending cycles. The bending cycles were carried out to determine the stability and reproducibility of the responses of the sensors. The bending of these sensors was also carried out in the same direction as that of the bending radii.

**Figure 7:**
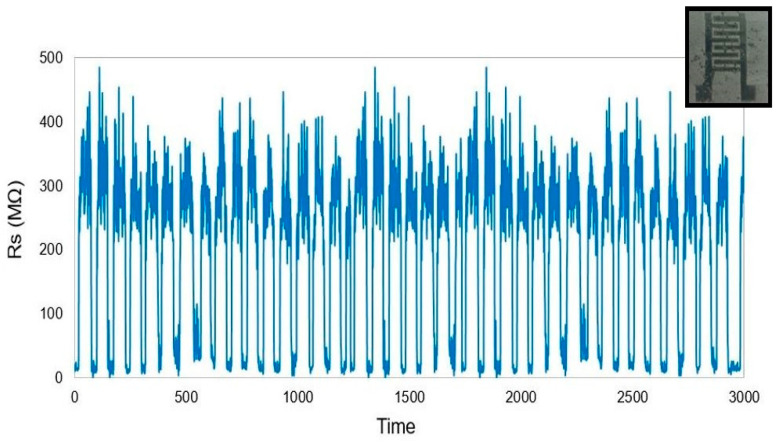
Response of the sensors for continuous bending cycles.

It is seen from Figure 7 that the output of the sensors was within a range of resistance values. The testing was done for a time period of over 20 min, where the strain level was kept as constant as possible for each cycle. The manual bending procedure made it challenging for the human hand to maintain an exact strain value, as can be reflected from the resistance values in Figure 7. The slight variations noticed in the responses between two testing cycles were within the error limit (±5%). However, there was a consistency in the changes in resistance values with each cycle, which marked the stability of the responses. The sensors were also durable in nature as the presence of two types of conductive nanomaterials increased their overall integrity. The sensors were further used for tactile sensing applications, where two different conditions were monitored to detect the potential of the sensors. Due to their operating principle relying on the tunnelling mechanism, these sensors have provided the additional advantage of tunability and customization of their sensitivity with respect to their composition. These prototypes are also much more stretchable and robust as compared to the other types of tactile sensors.

The biocompatibility of the processed materials makes these sensors an easy option for wearable sensing applications, where they can be embedded with conditioning circuits for real-time operations. The simple construction and fast response of these sensors make them a favourable choice over the magnetic tactile sensors. The high susceptibility to magnetic interference and noise also makes the magnetic tactile sensors unstable in a wide range of environmental conditions.

**Figure 8:**
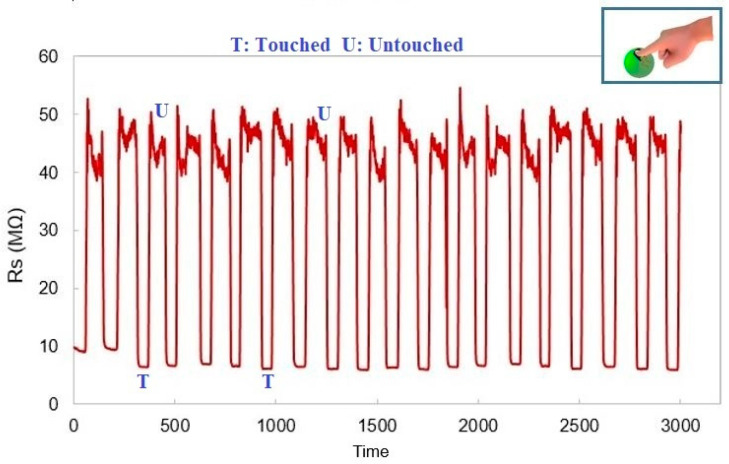
Response of the prototypes for tactile sensing. The inset figure shows the exertion of pressure on the sensing area of the prototypes.

Since the responses of these sensors are not affected by the ambient temperature, their sensitivity is more accurate as compared to the capacitive tactile sensors. The long-term stability in responses increases their credibility over optical sensors, the output of which is affected by the interference of the ambient light as well. Two different conditions of being ‘touched’ and ‘untouched’ were analyzed, referring to the contact and non-contact situations. A forward perpendicular direction was maintained to exert pressure on the sensors, thus bending them in a compressive direction. The entire process was conducted while wearing a glove in order to avoid any change in response due to the temperature or the permittivity of the skin. A constant pressure of ≈10 kPa was exerted on the sensing area of the electrodes. It is seen from Figure 8 that prompt and stable changes are apparent with respect to the applied pressure. An offset resistance value was present because the sensors were connected to a bent template (ball) for tactile operations. When the sensing area was touched, a shunt resistance was formed during the contact, thus decreasing the resistance and consequently increasing the current.

Further work related to the development of nanocomposites with CNTs could be carried out with the recent approaches involving compounds consisting of metal ions and organic ligands, called metallic-organic frameworks (MOFs) [62]. The presence of certain cross-linkers like polycarbazole along with CNTs can improve the overall synergy of the prototypes. These CNTs/MOFs have been able to generate a wide variety of composite-based sensors that are superior in terms of electromechanical characteristics as compared to their parent material [63]. Certain qualities like high porosity, tunable pore size and large surface area have allowed these prototypes to be used for applications in important fields. Along with tactile sensing [64], these sensors are highly efficient in electrochemical sensing applications where they are targeted to detect multiple ions with low detection limits. The presence of these MOFs causes certain attributes like excellent electron conductivity, stable polymerization, and minimization of the need for high-cost chemicals and construction facilities.

## 5. Conclusions

The paper exhibits the work done on fabricating and characterising the surfactant-induced MWCNTs/PDMS-based thin-film sensors. Doping of MWCNTs/PDMS nanocomposites was done using the SDS to increase the homogeneity and, subsequently, the electromechanical attributes of the sensors. The optimized values of 0.6 wt. % and 0.5 wt. % of MWCNTs and SDS, respectively, were used to mix with the PDMS matrix. The presence of MWCNTs and SDS with PDMS helped achieve high interfacial bonding of the nanocomposites. 

Some of the advantages of these prototypes include their low fabrication cost, simple operating principle, high flexibility, high stability and reproducibility of the responses, and the ability to be used as tactile sensors. The nanofillers followed the tunnelling mechanism within the polymer matrix to achieve enhanced connectivity. The prototypes had Young’s modulus and response time of 2.06 MPa and 1 millisecond, respectively. While the fracture point in their stress-strain behaviour was 68%, their response was stable and reproducible to a 40% strain value. During the low-pressure tactile sensing application, the sensors could detect a pressure of ≈10 kPa with high precision. The impedimetric responses of the prototypes showed that the change in the resistance values was consistent with the touch and no-touch situations. One of the issues faced during the experiments was the glitches in the oscillatory output. This could be due to the movement of wires and the slight inconsistency in the pressure exerted on them. These bottlenecks will be addressed in the next step, in addition to further experiments that are attached to signal-conditioning circuits to form a fully functionalized system for real-time wearable sensing applications.

## Figures and Tables

**Table 1:** Amount of CNTs and SDS mixed in the nanocomposites.

Carbon Nanotubes (wt. %)	Sodium Dodecyl Sulfonate (wt. %)								
0.3	0.1	0.15	0.2	0.25	0.3	0.4	0.45	0.5	0.55
0.6	0.1	0.15	0.2	0.25	0.3	0.4	0.45	0.5	0.55
0.9	0.1	0.15	0.2	0.25	0.3	0.4	0.45	0.5	0.55
1.2	0.1	0.15	0.2	0.25	0.3	0.4	0.45	0.5	0.55

## Data Availability

Not applicable.
